# Adjuvant intensity modulated whole-abdominal radiation therapy for high-risk patients with ovarian cancer FIGO stage III: final results of a prospective phase 2 study

**DOI:** 10.1186/s13014-019-1381-2

**Published:** 2019-10-21

**Authors:** Nathalie Arians, Meinhard Kieser, Laura Benner, Nathalie Rochet, Lars Schröder, Sonja Katayama, Klaus Herfarth, Kai Schubert, Andreas Schneeweiss, Christof Sohn, Katja Lindel, Jürgen Debus

**Affiliations:** 1 Department of Radiation Oncology, Heidelberg University Hospital, Im Neuenheimer Feld 400, D-69120 Heidelberg, Germany; 2 Heidelberg Institute of Radiation Oncology (HIRO), Heidelberg, Germany; 3 National Center for Tumor diseases (NCT), Heidelberg, Germany; 4 Institute of Medical Biometry and Informatics, University of Heidelberg, Heidelberg, Germany; 5 Department of Obstetrics and Gynecology, Klinikum Hanau, Academic Teaching Hospital of the Medical Faculty of the Goethe University of Frankfurt/Main, Hanau, Germany; 6 Clinical Cooperation Unit Radiation Oncology, German Cancer Research Center (DKFZ), Heidelberg, Germany; 7 Department of Radiation Oncology, Heidelberg Ion-Beam Therapy Center (HIT), Heidelberg University Hospital, Heidelberg, Germany; 8 German Cancer Consortium (DKTK), partner site, Heidelberg, Germany; 9 Department of Gynecology and Obstetrics, Heidelberg University Hospital, Heidelberg, Germany; 10 Department of Radiation Oncology, Municipal Hospital Karlsruhe gGmbH, Karlsruhe, Germany

**Keywords:** Ovarian cancer, Whole abdominal radiotherapy, Consolidation treatment, Toxicity, Quality of life, Oncological outcome

## Abstract

**Background:**

To assess late toxicity, quality of life and oncological outcome after consolidative whole abdominal radiotherapy (WART) following cytoreductive surgery and carboplatin/paclitaxel chemotherapy in high risk patients with advanced ovarian cancer FIGO stage III using IMRT (Intensity modulated radiation therapy).

**Methods:**

The OVAR-IMRT-02 study is a multi-center single-arm phase-II-trial. Twenty patients with optimally debulked ovarian cancer stage FIGO III with complete remission after chemotherapy were treated with intensity modulated WART. A total dose of 30 Gy in 20 fractions was applied to the entire peritoneal cavity. Primary endpoint was treatment tolerability; secondary objectives were acute and chronic toxicities, quality of life, rates of therapy disruption/abortion, progression-free survival (PFS) and overall survival (OS).

**Results:**

All patients completed treatment and 10/20 patients (50%) reached the final study follow-up of 36 months. Late side effects consisted of °1-°2 lower limb edema (44.5%), with one patient (5.6%) showing °3 edema. Three patients (16.7%) showed elevated gamma-Glutamyltransferase. There were no severe late side effects regarding renal or hepatic function or any gastrointestinal toxicity greater than °2. During WART, mean global health status decreased by 18.1 points (95%-CI: 7.1–29.0), but completely normalized after 6 months. The same trend was observed for the function scale scores. Kaplan-Meier-estimated 1-, 2- and 3-year PFS was 74, 51 and 40%, respectively. 1-, 2- and 3-year OS was 89, 83 and 83%, respectively.

**Conclusions:**

Intensity modulated WART after aggressive surgery and carboplatin/paclitaxel chemotherapy is associated with an acceptable risk of acute and late toxicity and minor impact on long-term quality of life. Together with the promising results for PFS and OS, intensity modulated WART could offer a new therapeutic option for consolidation treatment of patients with advanced ovarian cancer.

**Trial registration:**

The study is registered with ClinicalTrials.gov (NCT01180504). Registered 12 August 2010 – retrospectively registered.

## Background

With 295,414 new cases diagnosed in 2018, ovarian cancer accounts for 1.6% of all malignancies and 1.9% of all cancer-associated deaths worldwide [1]. Attempts for a better screening and thus early diagnosis or even prevention of ovarian cancer failed so far. Most women (75%) present with advanced-stage disease (FIGO stage III) or even distant metastases (FIGO stage IV) [2, 3]. Approximately 75–80% of patients with advanced stage ovarian cancer relapse after a median interval of 18–24 months [4], the abdominal cavity being the main location of recurrence. Overall survival (OS) decreases significantly in FIGO stage III and IV with 5-year-OS rates of only 31 and 13%, respectively [2, 3].

First-line treatment of advanced ovarian cancer patients consists of radical cytoreductive surgery (“no residual tumor”) and a platinum- and taxane-based chemotherapy (6 cycles of platinum-based chemotherapy in combination with paclitaxel 175 mg/m^2^) [5–18]. Simultaneous followed by consolidative administration of bevacizumab for 12–15 months can be considered in stage IIIB-IV patients with high risk for recurrence, accepting reduced quality of life [19–21].

In recent years many trials evaluated potential drugs to improve treatment for advanced ovarian cancer patients, aiming to prolong survival. PARP inhibitors represent one novel therapeutic option which is being investigated in several trials. To date, the greatest benefit has been in the maintenance setting, prolonging the progression-free survival of ovarian cancer patients with a BRCA1/2 mutation [4, 22–25].

Despite new technical developments in the field of radiation therapy in the last decades, consolidative radiotherapy of the peritoneal cavity has not been re-evaluated ever since it has been abandoned from standard treatment many years ago due to its severe side effects. Toxicity of WART using conventional radiation therapy has been widely investigated in the last decades [26–33], showing high toxicity and low therapy compliance rates. On the other hand, promising results for improved Progression-free Survival after WART have been reported. For example, significantly improved disease-free survival has been described for patients receiving surgery and radiation therapy compared with surgery and chemotherapy in a retrospective case-control study [31]. Furthermore, consolidation radiation therapy after resection and chemotherapy was associated with significantly prolonged relapse-free interval and OS. In this study, patients with complete remission after chemotherapy seemed to benefit most from consolidative radiation therapy. However, also in patients with microscopically residual disease, radiation treatment seems to be an efficacious treatment option [34]. Taking all these findings into consideration, WART may play a role as consolidation treatment in patients with advanced ovarian cancer showing macroscopically complete response after surgery and chemotherapy. Nowadays, using new technical developments like intensity modulated radiation therapy (IMRT), it is possible to reduce toxicity by sparing organs at risk (OARs) [35, 36]. The OVAR-IMRT-01 trial already showed the clinical feasibility of WART using IMRT [37]. Considering these promising results, we initiated a phase 2 study for further evaluation of treatment tolerance: the OVAR-IMRT-02 trial [38]. The primary objective of this study was to show a better treatment tolerance of WART using an IMRT technique compared with historical patient collectives receiving conventional radiation therapy. Preliminary data regarding treatment tolerability, acute toxicity and quality of life have already been published in 2017 [39]. Now we report the final results of late toxicities and quality of life, as well as PFS and OS.

## Methods

### Study design

The OVAR-IMRT-02 study is a multi-center single-arm phase-II-trial (investigator-initiated trial). Between 2010 and 2015, 20 patients with FIGO stage III ovarian cancer were treated with WART using IMRT with a dose of 30 Gy (daily fractions of 1.5 Gy 5 times per week) within a consolidation concept. Patient characteristics have already been described previously [39] and are shown in Table 1. All patients had maximal cytoreductive surgery (including at least total abdominal hysterectomy, bilateral adnexectomy, omentectomy, debulking of tumor masses) with postoperative residual tumor of < 1 cm followed by chemotherapy. Consolidative WART should not start later than 10 weeks after the last cycle of chemotherapy. Performance of IMRT using helical tomotherapy has been described previously [37, 38]. In short, WART was applied as helical IMRT using a tomotherapy device (TomoTherapy, Madison, WI) with 6-MeV photons. Control of positioning accuracy was performed daily with integrated megavoltage computed tomography for tomotherapy (3.5 MV). The clinical target volume included the whole peritoneal cavity extending from diaphragm to Douglas cavity, the liver surface, and the pelvic and para-aortic node regions. The planning target volume (PTV) encompassed an axial margin of 1.5 cm around the clinical target volume (2.5 cm in the cranial direction). Organs at risk (OARs) were kidneys, liver (except the 1-cm outer border), lungs, bones (vertebral bodies and pelvic bones), heart, and spinal cord. A dose of 30.0 Gy was prescribed to the median of the PTV. Inverse radiation dose planning was performed according to general recommendations (International Commission on Radiation Units and Measurements Report 50, 1999). There were no strict dose constraints for OARs used for optimization. The goal of optimization was to deliver a dose distribution in the PTV as homogeneous as possible, in addition to maximal sparing of OARs with a high priority on liver, kidney, and bone marrow. Tolerated maximum doses to OARs were not to exceed the tolerance dose 5/5 for each organ [25, 26]. Dosimetric information about the dose distribution in the target volume (PTV) and the OARs have already been reported previously [39].

**Table 1:** Baseline and clinical characteristics of the patients (*n* = 20)

Age (years)	58.4 ± 7.8
Karnofsky Index	
80%	5 (25%)
90%	14 (70%)
100%	1 (5%)
Size (cm)	163.6 ± 3.8
Weight (kg)	66.7 ± 11.7
Primary tumor localisation	
Ovary	16 (80%)
Fallopian tube	3 (15%)
Peritoneal	1 (5%)
Histology	
Serous	13 (65%)
Endometrial	4 (20%)
Others	3 (15%)
Tumor grade	
G2	3 (15.8%)
G3	16 (84.2%)
missing	1
Resection status	
R0	17 (85%)
R1	2 (10%)
R2 (< 1 cm)	1 (5%)
Excised lymph nodes	48.8 ± 25.2
Affected lymph nodes	3.4 ± 5.7
pN	
pN0	7 (35%)
pN1	12 (60%)
pN2	1 (5%)
Staging	
Ascites preoperatively	14 (70%)
Ascites postoperatively	3 (15.8%)
Affection of liver surface	2 (10%)
Peritoneal carcinosis	17 (85%)
bowel resection	4 (20%)
Tumor marker CA-125^a^	
Preoperatively	1625.7 ± 3567.0
	317.0 (89.0, 1369.7)
Postoperatively	160.7 ± 257.9
	61.9 (21.8, 187.8)
After chemotherapy	13.3 ± 7.4
	13.4 (7.0, 18.6)
Before start of radiation	14.5 ± 9.9
	13.4 (6.8, 20.1)
6 weeks after radiation	18.2 ± 17.5
	9.5 (5.8, 29.1)

^a^Due to the skewed distribution of tumor marker CA-125 also median, first and third quartiles are presented

Inclusion criteria were: histologically confirmed ovarian cancer stage FIGO III, maximal cytoreductive surgery (including at least total abdominal hysterectomy, bilateral adnexectomy, omentectomy, debulking of tumor masses) with postoperative residual tumor of < 1 cm (R0, R1, R2 with maximal diameter of largest tumor residual of 1 cm), adjuvant chemotherapy with complete remission after chemotherapy, Karnofsky performance score > 60, age > 18 years, and written informed consent.

Patients were followed for 36 months after WART. Follow-up visits were scheduled at 6 weeks and 3, 6, 9, 12, 15, 18, 24, 30, and 36 months after treatment. Each visit included update of case history, documentation of adverse events according to Common Terminology Criteria for Adverse Events (CTCAE) version 3.0, assessment of quality of life using the European Organization for Research and Treatment of Cancer QLQ-C30 questionnaire, gynecologic examination, transvaginal ultrasound, and a blood sample including tumor marker checks (CA- 125). In addition, pelvic-abdominal CT or MRI scans were performed 6, 12, and 24 months after treatment. The primary endpoint was treatment tolerance, defined as the lack of any CTCAE °4 toxicity. Secondary endpoints were rate of therapy disruption, rate of therapy abortion, acute toxicity (< 6 weeks after end of WART), chronic toxicity (> 6 weeks after end of WART), quality of life, PFS and OS. The study was approved by the local Ethics Committee of Heidelberg University. Written informed consent was obtained from each participant before entering the trial.

### Statistical analysis

The study was planned using an adaptive two-stage design allowing for a sample size recalculation after the interim analysis. Details on the applied design and sample size recalculation can be found in the article presenting the results on the short-term endpoints [39]. The first part included the confirmatory analysis, where the tested null hypothesis stated that the true rate of patients for whom no CTCAE °4 toxicity occurs during radiation therapy and until 6 weeks after its termination amounts to at most 70%. In this article, results on the secondary endpoints regarding follow-up data are presented. Different types of toxicities occurring 6 weeks after termination of WART are analyzed using absolute and relative frequencies. Quality of life is evaluated for each follow-up visit using mean and standard deviation. Kaplan-Meier method is used to estimate survival rates of PFS and OS. For PFS and OS, time was calculated from start of WART until disease recurrence or death. Statistical analyses were performed using the Statistical Analysis System, version 9.4 (SAS Institute, Cary NC), and figures were prepared in R, version 3.5.0 [40].

The study is registered with ClinicalTrials.gov (NCT01180504).

## Results

All patients completed WART according to the study protocol. Results of the primary endpoints have already been published, showing an observed tolerability rate of the study treatment of 95%. Ten of 20 (50%) patients completed the study follow-up of 36 months. Two patients withdrew the consent to participate in the trial. Five patients were lost to follow-up, one after 9 months and two after 12 and 24 months, respectively. Three patients had died in the course of the follow-up with the last available follow-up visit at 6 weeks, 6 and 12 months, respectively.

### Late toxicity analysis

**Fig. 1:**
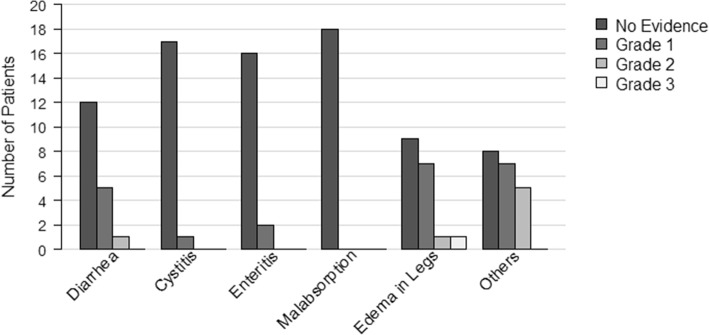
Late toxicities of intensity modulated WART. Incidence of late toxicities according to Common Terminology Criteria for Adverse Events version 3.0. (Maximal Common Terminology Criteria for Adverse Events grade later than 6 weeks after end of WART)

Late toxicities are shown in Fig. 1. Late side effects of intensity modulated WART mostly consisted of lower limb edema. Seven patients (38.9%) developed °1 edema, one patient (5.6%) °2 and °3 edema, respectively. No late gastrointestinal toxicities greater than °2 were observed. Five patients (27.8%) reported °1 and one patient (5.6%) °2 diarrhea, respectively. Two patients (11.1%) showed enteritic symptoms °1. One patient developed cystitis °1 (5.6%). Other side effects mostly consisted of fatigue °1-°2, constipation °1, nausea °1-°2, abdominal pain °1-°2, edema of the abdomen °1 and pruritus °1. For 3 patients, an ileus has been reported during follow-up, which was mainly associated with disease recurrence.

**Fig. 2:**
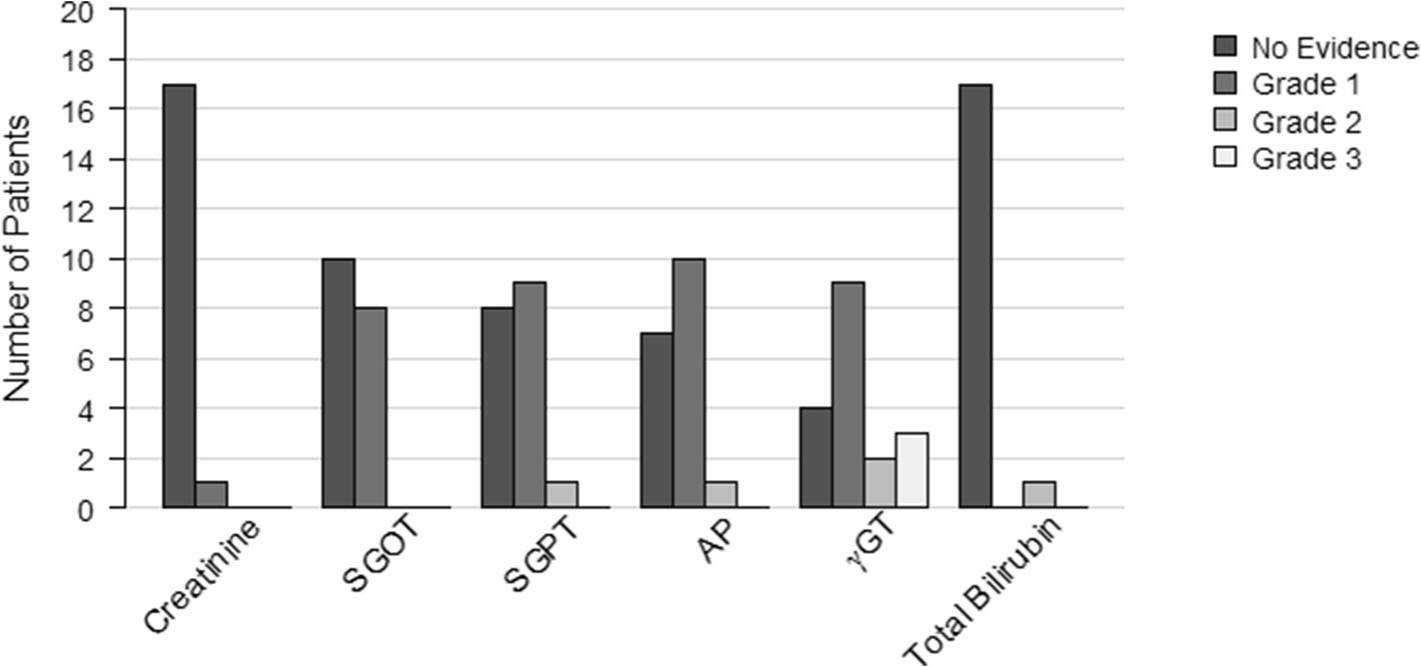
Late hematological toxicities of intensity modulated WART. Incidence of late hematologic toxicities according to Common Terminology Criteria for Adverse Events version 3.0. (Maximal Common Terminology Criteria for Adverse Events grade later than 6 weeks after end of WART). Abbreviations: AP = alkaline phosphatase; γGT = gamma-Glutamyltranferase; S-GOT = serum glutamic oxaloacetic transaminase; S-GPT = serum glutamic pyruvic transaminase

Hematological toxicities are shown in Fig. 2. There were no severe late side effects regarding renal or hepatic function. One patient (5.6%) showed elevated creatinine °1. In total, 12 patients showed slightly elevated liver enzymes with 8 patients (44.4%) showing elevated SGOT (serum glutamic oxaloacetic transaminase) °1, 9 patients (50%) and one patient (5.6%) showing elevated SGPT (serum glutamic pyruvic transaminase) °1 and °2, respectively. Three patients (16.7%) showed elevated gamma-Glutamyltransferase (γGT) °3, which is a parameter for cholestasis. Furthermore, 50%/11.1% of patients showed elevated γGT °1/°2 and 55.6%/5.6% of patients showed elevated alkaline phosphatase (AP) °1/°2, respectively. One patient (5.6%) was observed with isolated elevation of bilirubin °2.

### Analysis of long-term quality of life

**Fig. 3:**
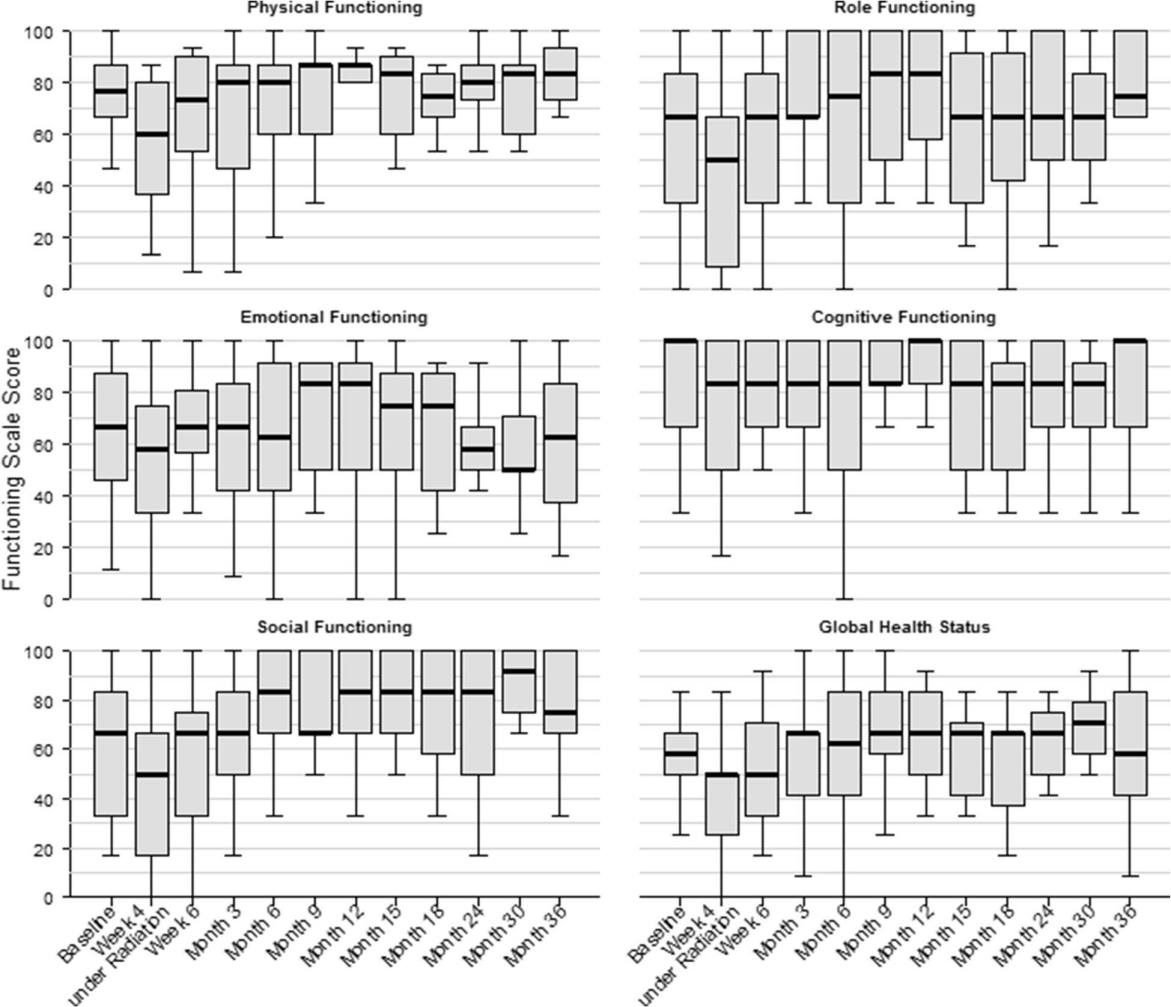
Analysis of quality of life. Function scale scores and global health status scores at baseline, week 4 during radiation therapy, 6 weeks and month 3, 6, 9, 12, 15, 18, 24, 30 and 36 after radiation therapy

Baseline function scale scores were available for all 20 patients; baseline global health status scores were available for 19 patients. Data for week 4 during and 6 weeks after WART and month 3, 6, 9, 12, 15, 18, 24, 30 and 36 were available for 19, 19, 18, 18, 17, 15, 12, 11, 9, 8 and 8 patients, respectively. Box plots of function scale scores and global health status scores at baseline and all follow-up visits are shown in Fig. 3. Patients presented with a mean global health status score of 57.9 +/− 15.6 at baseline. We could already show that the mean global health status score decreased by 18.1 points (95% CI 7.1–29.0) 4 weeks after starting WART [39]. However, 6 months after WART the score had normalized completely with a mean score of 57.9 +/− 26.3. The score even increased above baseline level during further follow-up with a maximum mean score of 69.8 +/− 14.7 after 30 months. Physical functioning score at baseline was 74.1 +/− 19.2. The lowest score could be observed 4 weeks after starting WART, with continuous increase back to baseline after 9 months with a mean score of 76.1 +/− 19.2, which even increased further with a maximum mean score after 36 months of 83.3 +/− 11.7. Role functioning score at baseline was 58.3 +/− 33.1. The lowest score could be observed 4 weeks after starting WART, with continuous increase back to normal already 6 weeks after WART with a mean score of 60 +/− 33.5. The maximum mean score of 75.6 +/− 25.1 was observed after 12 months. Emotional functioning score at baseline was 63.9 +/− 26.4, which decreased to a minimum mean score of 55.7 +/− 29.4 4 weeks after starting WART and showed an increase back to baseline level 6 weeks after WART with a mean score of 65.8 +/− 22.9. The maximum score could be observed after 9 months with a mean score of 71.6 +/− 22.3. Cognitive functioning score at baseline was 84.2 +/− 21.3. The lowest score was observed after 6 months with a mean score of 71.3 +/− 33.7, which increased nearly back to baseline after 36 months with a mean score of 83.3 +/− 25.2. Social functioning score at baseline was 61.7 +/− 26.5, which decreased to a minimum score of 43.9 +/− 36.1 4 weeks after starting WART, with continuous increase back to baseline after 3 months. The maximum mean score of 80.6 +/− 19.9 was observed after 15 months.

### Analysis of oncological outcome

Eleven patients (55%) experienced disease recurrence, all of them intraperitoneally. 8 (72.7%) of all first recurrences were localized inside the peritoneal cavity only, 3 (27.3%) were localized inside as well as outside. Different patterns of intraperitoneal recurrence were observed: 2 (18.2%) patients presented with malignant ascites only, 5 (45.5%) with a new macroscopic tumor lesion and 3 (27.3%) with a combination of both. One patient (9.1%) developed a new macroscopic tumor lesion together with malignant pleural effusion. All in all, 7 patients showed up with distant metastases during follow-up. Details of recurrence patterns and salvage treatment are listed in Table 2. Partial remission after salvage therapy of first recurrence could be achieved in 5 cases, 3 patients showed progressive disease and 4 patients received further second line therapies because of progression in the course of the follow-up.

**Table 2:** Patterns of recurrence and salvage treatment

Site of recurrence														
			Distant metastases							Salvage treatment				
Pat.	Number of recurrence	Peritoneal carcinomatosis	LR	HEP	PUL	pleural	MED	SPL	CER	CTX	ITX	Targeted TX	OP	others
1	1	x		x						x				
2	1	x			x	x				x	x			
3	1	x								x				
3	2	x		x						x				
5	1	x							x	x				x
7	1													x
8	1									x	x			
8	2			x			x			x				
11	1	x												
11	2	x	x							x				x
11	3	x		x	x			x			x	x		
11	4	x		x			x			x	x			
11	5	x		x			x	x		x				
13	1													
16	1	x	x							x			x	
18	1	x		x						x				
19	1	x								x			x	
19	2	x	x			x							x	x

*Pat* patient, *LR* local recurrence, *HEP* hepatic, *PUL* pulmonal, *MED* mediastinal, *SPL* splenic, *CER* cerebral, *CTX* chemotherapy, *ITX* immunotherapy, *TX* therapy, *OP* operation

**Fig. 4:**
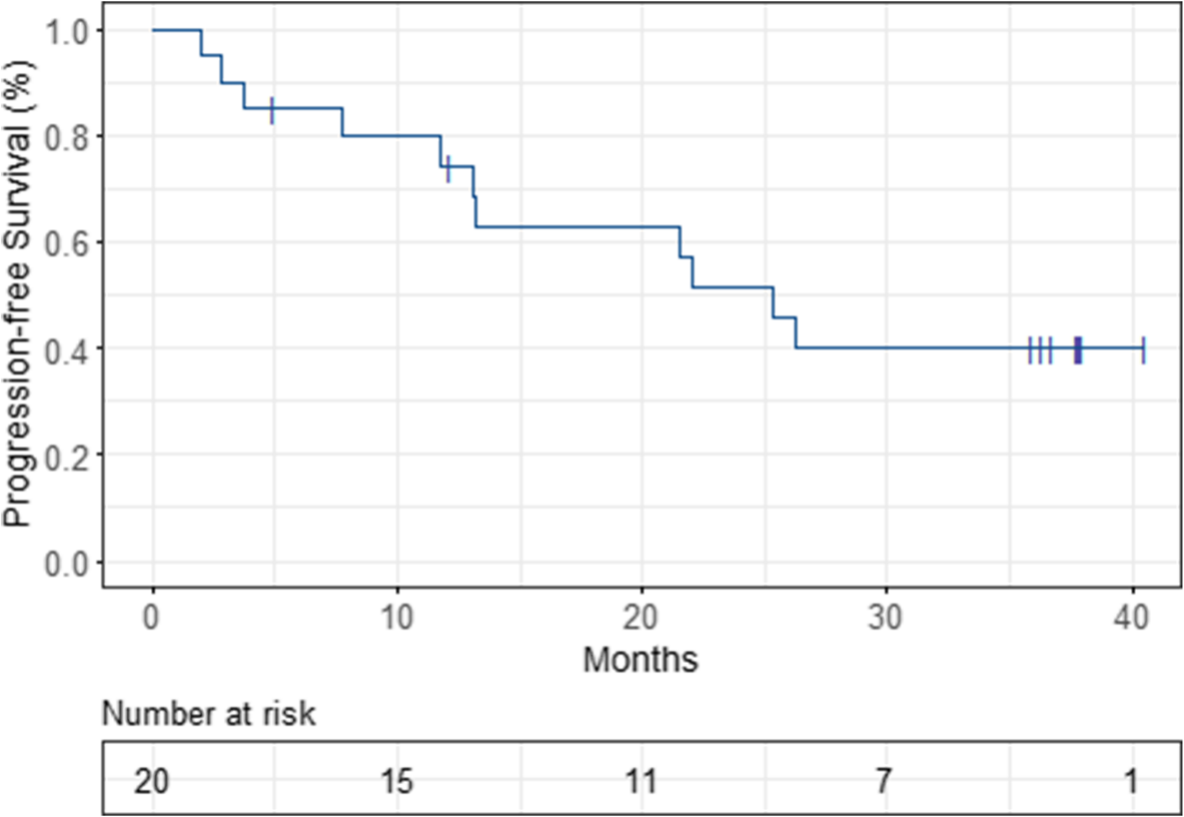
Kaplan-Meier-estimated Progression-free Survival (PFS)

**Fig. 5:**
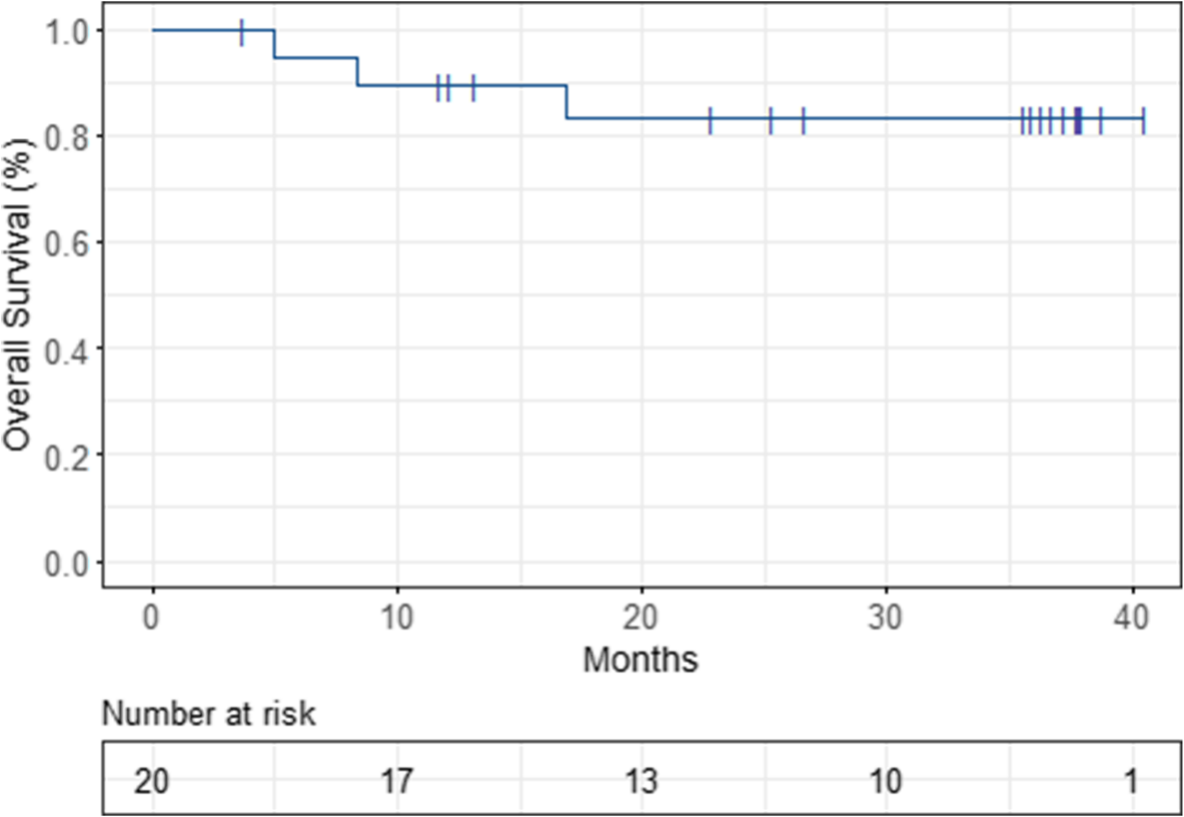
Kaplan-Meier-estimated Overall Survival (OS)

Median time to recurrence was 25.3 months. Estimated 1-, 2- and 3-year-PFS was 74, 51 and 40%, respectively (Fig. 4). Three patients died during follow-up after 5, 8.3 and 16.9 months, respectively. Two of them died because of disease progression, 1 patient died because of operative complications. Ten patients (50%) were known to be alive at the end of the study, 7 patients were censored in the course of the trial. Estimated 1-, 2- and 3-year-OS was 89, 83 and 83%, respectively (Fig. 5).

## Discussion

These data confirm the previously reported excellent tolerability rate of intensity modulated WART with no clinically relevant severe acute or late side effects. The clinical feasibility of intensity modulated WART has already been shown in the OVAR-IMRT-01 trial. The use of IMRT resulted in excellent coverage of the whole peritoneal cavity and effective sparing of OARs [37]. Furthermore, the OVAR-IMRT-02 trial showed excellent treatment tolerability of intensity modulated WART with an observed tolerability rate of 95%. Treatment could be completed in all cases without any toxicity-related treatment interruption [39]. Only 1 acute CTCAE °4 toxicity was observed (thrombocytopenia). No acute nor late gastrointestinal, hepatic or renal toxicities greater than °2 have been observed [39]. Late side effects of intensity modulated WART mostly consisted of lower limb edema °1-°2, with only one patient showing °3 edema. Laboratory signs of °3 cholestasis with elevated γGT could be observed in 3 patients, without any clinical relevance. Analysis of patient-reported quality of life showed decreased quality of life during WART but also a very quick recovery after treatment completion within 6 months after WART. The score even increased above baseline level with a maximum score after 30 months. Function scale scores showed similar characteristics, showing full recovery between 6 weeks and 9 months after completion of WART and showing even higher scores compared to baseline during follow-up. However, these findings have to be interpreted with caution. As patients with progression and death aren’t represented in the scores at later time points of the follow-up, the scores might be estimated too high. Only the cognitive function scale score increased only slowly and reached baseline levels at the end of study at 36 months.

Prior studies have shown that WART can be curative in certain groups of patients [41, 42]. Our study showed an estimated 1-, 2- and 3-year-PFS of 74, 51 and 40%, respectively. Median time to recurrence was 25.3 months, which is very good compared to other trials. For example, the ICON7 trial reported a median PFS of 19.9 months after administration of chemotherapy + bevacizumab, which even dropped to 16 months in the high-risk collective [21]. 3-year-OS of our cohort was also relatively good with 83%. Informative value of this statistical analysis is very limited due to the small sample size of 20 patients, of which only 10 patients (50%) completed the study follow-up of 36 months and of which only 3 patients experienced death.

One promising development within the scope of establishing better treatment strategies for advanced ovarian cancer patients was the introduction of bevacizumab into treatment [19, 20]. In 2011, a phase 3 study reported significantly prolonged median PFS by the use of bevacizumab during and up to 10 months after carboplatin/paclitaxel chemotherapy in patients with advanced ovarian cancer [20]. In the updated analysis in 2015, PFS of the entire population was no longer statistically significant and there was no impact on OS [21]. Furthermore, the addition of bevacizumab was associated with side effects like hypertension (22.9%) and gastrointestinal wall disruption (2.6%) requiring medical therapy [20]. PARP inhibitors represent another novel therapeutic option, especially for BRCA-mutated ovarian cancer patients. The SOLO-1 trial investigated olaparib as maintenance at the completion of first-line chemotherapy in FIGO stage III–IV, BRCA-mutated ovarian cancer patients, showing a substantial PFS-benefit [24]. Serious adverse events occurred in 21% with anemia (7%) being the most common event. There are also trials evaluating combined treatment with both bevacizumab and olaparib, for example the ongoing PAOLA-1 trial.

Despite all these achievements, the main reason for progression and death is still recurrence inside the peritoneal cavity. This fact suggests that more locally aggressive treatment regimens are necessary to improve PFS and OS rates of patients with advanced ovarian cancer being at high risk for recurrence. In that context, intensity modulated WART is a promising therapeutic option for consolidation treatment. Further investigations are necessary, evaluating the potential of WART, also in combination with any of the above mentioned substances.

## Conclusions

Intensity modulated WART is a promising treatment option for all advanced-stage ovarian cancer patients with excellent treatment tolerance, acceptable acute and late toxicities and only minor impact on long-term quality of life. Further randomized trials are necessary to further evaluate the promising PFS and OS rates observed in the OVAR-IMRT-02 trial. Additionally, the combination of WART with PARP inhibitors and bevacizumab should be evaluated.

## Data Availability

The datasets generated and/or analysed during the current study are not publicly available due personal data protection reasons of the study participants but are available from the corresponding author on reasonable request.

## Abbreviations

CT: Computed tomography
CTCAE: Common Terminology Criteria for Adverse Events
FIGO: Fédération Internationale de Gynécologie et d‘Obstétrique
Gy: Gray
IMRT: Intensity Modulated Radiation Therapy
MeV: Mega electron volt
MRI: Magnetic resonance imaging
OAR: Organ at risk
OS: Overall survival
PARP: Poly ADP ribose polymerase
PFS: Progression-free survival
PTV: Planning target volume
SGOT: Serum glutamic oxaloacetic transaminase
SGPT: Serum glutamic pyruvic transaminase
WART: Whole abdominal radiotherapy
γGT: gamma-Glutamyltransferase
